# The impact of regional screening policies on the diffusion of cancer screening participation in Belgium: time trends in educational inequalities in Flanders and Wallonia

**DOI:** 10.1186/s12913-018-3746-x

**Published:** 2018-12-04

**Authors:** Barbara Willems, Piet Bracke

**Affiliations:** Health and Demographic Research, Department of Sociology, Ghent University, Korte Meer 5, 9000 Ghent, Belgium

**Keywords:** Educational inequalities, Diffusion of innovation theory, Cancer screening participation, Regional screening policies

## Abstract

**Background:**

We investigate whether the extent of educational inequalities in the use of Pap smears (cervical cancer screening) and mammograms (breast cancer screening) in Belgium has changed over time in accordance with the pattern predicted by diffusion of innovation theory, as well as how the regional cancer screening policies of Flanders and Wallonia influence this pattern.

**Methods:**

Data were obtained from five successive cross-sectional waves (1997–2001–2004-2008-2013) of the Belgian Health Interview Survey. Final sample sizes consisted of 8988 women aged 25–64 years for cervical cancer screening and 4194 women aged 50–69 years for breast cancer screening. We calculated absolute and relative measures of inequality, more specifically, the slope index of inequality (SII) and the relative index of inequality (RII), and their development over time.

**Results:**

In both Flanders and Wallonia, mammogram use increased greatly between 1997 and 2013, while Pap smear use has remained quite stable over time. Educational inequalities in cervical-cancer screening have been largely persistent over time in both regions. In contrast, educational inequalities in breast cancer screening fluctuated more between 1997 and 2013. Between 1997 and 2001, when the breast cancer screening programme was implemented in Flanders, RII reduced significantly by 45%. Inequality measures did not change significantly in Wallonia, where it is known that most women are screened opportunistically outside the programme.

**Conclusions:**

By focussing on Belgium, this study demonstrates that regional variations in the support of a national screening programme can result in regional variations in the pattern of diffusion for cancer screening, as well as to the development of inequalities in cancer screening participation. Moreover, the findings demonstrate that high visibility and awareness of the screening programme, as was more the case in Flanders than it was in Wallonia, are required in order to reduce or eliminate educational inequalities in cancer screening participation over time. General practitioners and gynaecologists can play a decisive role in this regard.

## Background

Research has focused primarily on the facilitators and barriers associated with participation in cancer screening, as well as on socioeconomic inequalities in the uptake of screening tests [1–9]. Factors associated with the gradual spread of cancer screening tests within a population over time have received far less attention [10]. As a result, developments in socioeconomic inequalities in the uptake and spread of screening tests over time remain unclear. *Diffusion of innovation* (DOI) theory offers a useful perspective for studying such universal processes of social change. As suggested by previous studies on health innovations, DOI theory can be applied to cancer screening behaviour as well [10, 11].

According to DOI theory, new preventive technologies or health interventions (e.g. cancer screening tests) spread through a population in a predictable pattern resembling an S-shaped curve [12, 13]. When a cancer screening test is first introduced, only a few people will adopt the innovation. In other words, only a few people will use the screening test (these are the ‘early adopters’). Later, as the rate of uptake accelerates and enough people use the test (the ‘early majority’), the screening will gain critical mass and become increasingly widespread through the population. Finally, the increase in uptake will decelerate, as fewer and fewer remaining potential members of the population (the ‘late majority’ and ‘laggards’) will use the test. According to DOI theory, the various categories of adopters are strongly associated with socio-economic position [12, 13]. In fact, early adopters tend to have a higher social status and more years of education than do members of the late majority or the laggards. Consequently, the diffusion of an innovation follows a cycle of adoption in which, over time, the innovation spreads through a population from those with the highest level of education to those with the lowest. As posited by the *inverse equity hypothesis,* the first stage of the diffusion – in which a cancer screening test has recently been introduced – is generally accompanied by a widening gap in use between people with high and low levels of education, as the test is likely to reach those with higher levels of education first [14]. Only in a further diffusion stage, when the more highly educated groups have reached threshold levels of use and those with lower educational attainment gain greater access to the screening test, will the initially increasing educational gap once again start to shrink. In summary, rates of participation in cancer screening, as well as the associated educational inequalities in use, tend to fluctuate over time along an S-shaped curve.

Although previous research has consistently found that women with higher levels of education are more likely to participate in screening for cervical and breast cancer, as compared to their counterparts with less education [3, 5–7, 15–17], less attention has been paid to how such educational gaps in cancer screening participation have developed over time. In addition, with a few exceptions [10, 18, 19], DOI theory has not been applied as a framework within which to explain time trends in the unequal participation in cancer screening. As result, cancer screening researchers have tended to overlook the fact that different screening tests, which have been introduced into clinical practice at different points in time, are positioned at different stages in the diffusion process, which are thus associated with different levels of inequality [19]. Moreover, existing studies fail to address the impact of cancer screening policies or strategies on the diffusion pattern.

In general, cancer screening strategies can be categorised as either ‘organised’ or ‘opportunistic’. In organised screening, screening activities are carried out as part of an organised programme, which requires [1] the active and systematic identification and invitation of a defined target population (i.e. the eligible screening population); [2] the use of homogeneous criteria and quality-control activities and [3] the evaluation of results and quality [20–22]. In contrast, in opportunistic screening, screening tests are offered only in primary or other healthcare settings. Their use thus depends on the spontaneous initiative of patients and their physicians. Given that the opportunistic approach does not systematically identify and invite the eligible screening population, it is more likely to result in variability in those who participate, as well as in the quality of the screening process. Cancer screening strategies can differ both between and within countries, depending on the type of cancer being screened (e.g. breast, cervical).

Belgium offers an interesting case with which to study the impact of screening strategies (organised vs. opportunistic) on the diffusion patterns of cancer screening tests. First, given that different screening strategies are applied for the screening of breast and cervical cancer in Belgium, variations in usage according to screening strategy might be visible. Second, given the clear differences between Belgian regions with respect to the organisation of cancer screening, regional variations might occur even when the same screening strategy is used. More specifically, the gradual cultural and social divergence between the Dutch-speaking northern part (Flanders) and the French-speaking southern part (Wallonia) of Belgium gave rise to two separate political systems. Within this complex political system, regional authorities are charged with the organisation, management and evaluation of cancer screening, with the Federal state retaining responsibility primarily for the financing of most medical acts and reimbursements. This situation has generated regional differences in terms of organisation, management, promotional activities, culture and prescription behaviour [4].

In Belgium, breast cancer screening by means of mammography (the process of using low-level X-rays to examine the human breast for diagnosis and screening) was introduced in the 1970s [23]. In 1987, the introduction of the EU programme ‘Europe against Cancer’ resulted in a generalisation of screening campaigns, and the increased control associated with this programme enhanced the homogeneity and quality of mammography [24]. Until 2000, however, breast cancer screening in Belgium lacked any consistent policy or strategy [25]. In fact, municipalities, provinces, and the Flemish and Belgian governments independently carried out programmes to raise awareness about the importance of breast cancer screening without proper coordination between such programmes. Since 2001 in Flanders and since 2002 in Brussels and Wallonia, breast cancer screening has been organised at the population level in a national breast cancer screening programme, in line with the European guidelines for quality assurance. In this programme, every eligible woman between the ages of 50 to 69 years receives a personalised letter every two years with a set appointment for a mammogram, which is paid entirely and directly by the healthcare insurance to the mammography unit [26]. In other words, the mammogram is provided free of charge. Despite the nationwide implementation and organisation of the screening programme, a wide gap exists between the Belgian regions [27]. In Flanders, half of all women between the ages of 50 and 69 years have had a mammograms through the organised programme. In Wallonia and Brussels, most women are screened outside the organised programme during consultations with their general practitioners or gynaecologists. Efforts to organise a national screening programme notwithstanding, the opportunistic screening of breast cancer remains the most common way of screening in the south of Belgium.

Despite EU [28] recommendations that cancer screening should be offered only in organised, population-based programmes with quality assurance at all levels, cervical cancer screening in Belgium is not population-based, and it remains essentially opportunistic. Since 1965, specialised mobile teams or fixed centres have organised periodic screening in Flanders. Due to low participation rates, however, this vertical system was abandoned in the early 1980s and replaced by a gradual increase in opportunistic screening by private gynaecologists and, to a lesser extent, by general practitioners [29]. Although screening initiatives were set up in the Flemish provinces, efforts to start a central cervical cancer screening programme have thus failed so far [27]. In addition, the opportunistic cervical screening in Belgium is characterised by a high level of over-screening (i.e. the model screening interval is one year, instead of the European guideline of three years) [27, 30]. Moreover, as is generally the case in the context of over-screening, over-consumption has emerged only in one part of the eligible population, while under-consumption has occurred in the other part. More specifically, women with high socio-economic status are screened more than necessary, while older and more socio-economically disadvantaged women are particularly likely to remain unscreened [30, 31]. To address over-consumption, the governments have gradually taken steps to reduce the reimbursement of cervical cancer screening or ‘Papanicolaou’ tests (also known as ‘Pap smears’). Prior to 2009, Pap smears were reimbursed once every year. Between 2009 and 2013, they were reimbursed once every two years and, since 2013, they have been reimbursed once every three years [27]. In contrast to mammogram use, regional differences in Pap smear use are less pronounced.

Government policies (e.g. the introduction of an organised screening programme) can influence the diffusion pattern and unequal use of cancer screening tests [10]. More specifically, the implementation of a national population-based programme for breast cancer screening in 2001–2002 might have reshaped the diffusion pattern of mammogram use in Belgium. Screening programmes are aimed at addressing the drawbacks of individual decision-making in opportunistic screening by upscaling the screening process to the population level. Benefits of this strategy include the systematic identification and invitation of the population group at risk, specific appointments, higher quality and full reimbursement of the screening costs. Because of these benefits, population-based programmes are better able to reduce the education gradient in the use of cancer screening tests [6, 7, 9, 20, 32, 33]. In other words, such screening programmes can respond to and reshape the classic diffusion pattern predicted by DOI theory. Previous studies by Puddu and colleagues [4] and by Renard and colleagues [5] have investigated whether the introduction of the Belgian national programme for breast cancer screening reduced educational inequalities in mammogram use over the periods 2001–2004 and 1997–2008, respectively. These studies demonstrate that the screening programme improved mammogram use by Belgian women, although it did not completely counteract the education gradient in use. Although the results of these two studies do reveal a decreasing education gradient over time, the authors note that this decline had apparently started before the introduction of the programme. Because these studies do not distinguish between regions, however, they are unable to explore the possible role of the high rate of screening outside the programme in the regions Brussels and Wallonia in the observed persistence of educational inequalities in mammogram use.

The purpose of the present study is to investigate the impact of regional screening policies in Belgium on the diffusion patterns of two different screening tests: Pap smears (for cervical cancer screening) and mammograms (for breast cancer screening). Both of these tests target women, and both are characterised by relatively high usage rates in Belgium [5, 26, 34, 35]. The screening strategies applied for the two tests differ, however, with cervical cancer screening being opportunistic, while breast cancer screening is organised through a screening programme. More specifically, this study focuses on how educational inequalities in the use of mammograms and Pap smears have developed over time, relative to the pattern predicted by DOI theory and whether this pattern varies according to regional differences in screening policies.

## Methods

### Study population and data

Data were obtained from five successive waves (1997, 2001, 2004, 2008 and 2013) of the Belgian Health Interview Survey (BHIS). The repeated cross-sectional design and consistent methodology (e.g. composition of the sample, organisation of fieldwork) of the BHIS make it possible to assess trends across the various years of the survey and to evaluate the impact of the implementation of the national breast cancer screening programme in 2001–2002 [36]. Households and their members were selected from the National Register following a multi-stage stratified sampling procedure. The information was collected through face-to-face interviews, as well as through a self-administered questionnaire. A complete description of the methodological foundation and evolution of the BHIS has been published by Demarest and colleagues [36]. In the current study, we adopted the final sample sizes according to the European guidelines for the eligible screening population for each cancer type: women between the ages of 25 and 64 years for cervical cancer screening, and women between the ages of 50 and 69 years for breast cancer screening. We omitted all cases with missing information (*N* = 754; 5.6%), as well as cases having cancer at the time of the interview (*N* = 246; 1.9%). This results in final samples of 8988 women for cervical cancer screening (*N* = 4405 in Flanders, *N* = 4583 in Wallonia) and 4194 women for breast cancer screening (*N* = 2075 in Flanders, *N* = 2119 in Wallonia). Data from Brussels were excluded from our study, as the sample sizes per wave were too small to provide meaningful analyses of inequalities across educational groups.

### Model variables

#### Dependent variables

Self-reported mammogram and Pap smear use were considered as the dependent variables. Women were asked whether they had undergone a mammogram in the past two years and a cervical smear test in the past three years (‘yes’ or ‘no’). The questionnaire did not distinguish between screening, diagnostic and follow-up mammograms and Pap smears, nor between opportunistic or programme-based screening.

#### Main independent variable

Educational attainment was measured as the highest level of education completed. This information was recoded into four categories, according to the International Standard Classification of Education (ISCED 2011): 0 = no diploma/primary education, 1 = lower secondary education, 2 = higher secondary education, and 3 = tertiary education [37].

#### Potential confounding variables

As known from previous research, older women, women from ethnic minority groups, women living in rural areas, unemployed women and women with financial difficulties tend to engage less in screening [38–42]. We therefore controlled for age (categories vary across screening tests), country of birth (0 = Belgium, 1 = not Belgium), urbanisation (0 = urban, 1 = rural), employment status (0 = employed, 1 = not employed), necessity of postponing medical consumption (0 = no, 1 = yes) and difficulty contributing to healthcare (0 = not hard to pay, 1 = hard to pay).

### Statistical analyses

First, Pap smear coverage (the proportion of women aged 25–64 years who reported having had a Pap smear in the past three years) and mammogram coverage (the proportion of women aged 50–69 years who reported having had a mammogram within the past two years) was estimated for the five waves, broken down by region and educational level. In addition, we created a graphic display of the diffusion patterns of mammogram and Pap smear use between 1997 and 2013 in Flanders and Wallonia, overall and by educational attainment.

Second, to estimate the gradient of educational inequality in the use of mammograms and Pap smears, we adopted both relative and absolute regression-based measures of inequality that are recommended when making comparisons over time or across populations: the relative index of inequality (RII) and the slope index of inequality (SII) [43, 44]. Both of these measures offer the advantage of considering screening attendance in all educational levels, along with the relative position and size of each educational level within a population, instead of merely comparing the two most extreme groups. The measures are therefore able to take into account shifts occurring within the educational hierarchy when results are compared over time. They are calculated by transforming educational level into a summary measure that is scaled from 0 to 1 (representing the lowest and highest hypothetical level of education, respectively). To reflect the share of the sample at each educational level, the population in each education category is assigned a modified ridit score on the scale, based on the midpoint of the range in the cumulative distribution of the population within a given category. As suggested by Zou [45], we applied a ‘modified Poisson regression’ approach to compute RII and SII with 95% confidence intervals (CI), as this strategy offers a solution for the convergence issues associated with the binary approach and provides more robust estimates than the binary approach does [43, 46]. A RII greater than 1 and a positive SII imply that, compared to those with less education, women with higher levels of education are more likely to use the screening test. We created graphs to display the diffusion patterns of relative (RII) and absolute (SII) educational inequalities between 1997 and 2013 in Flanders and Wallonia.

Third, to assess the development of RII and SII over time, these estimates were compared between two different survey years using the interaction test, as applied in the method reported by Altman and Bland [47]. To calculate the test, the difference of the two log odds ratios or relative risks was divided by its standard error. The corresponding *p*-value of the test was obtained on the table of the z-distribution. All analyses were performed with STATA 13, in order to account for the multistage sampling design of the BHIS.

## Results

### Mammogram and Pap smear use in Flanders and Wallonia between 1997 and 2013

**Fig. 1:**
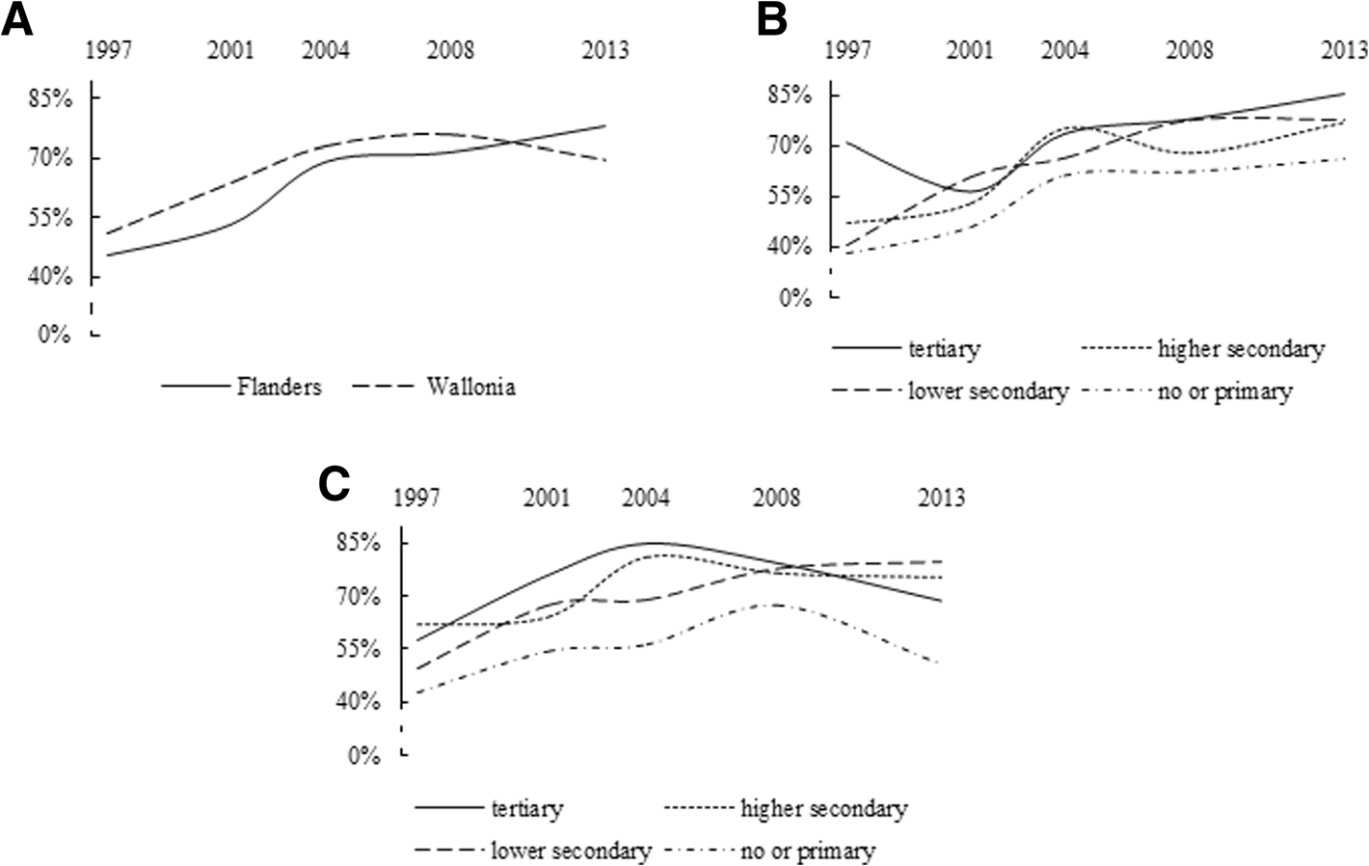
**a** Diffusion of mammogram use in women 50–69 years old between 1997 and 2013, by region. **b** Diffusion of mammogram use in women 50–69 years old between 1997 and 2013 in Flanders, by educational level. **c** Diffusion of mammogram use in women 50–69 years old between 1997 and 2013 in Wallonia, by educational level

**Fig. 2:**
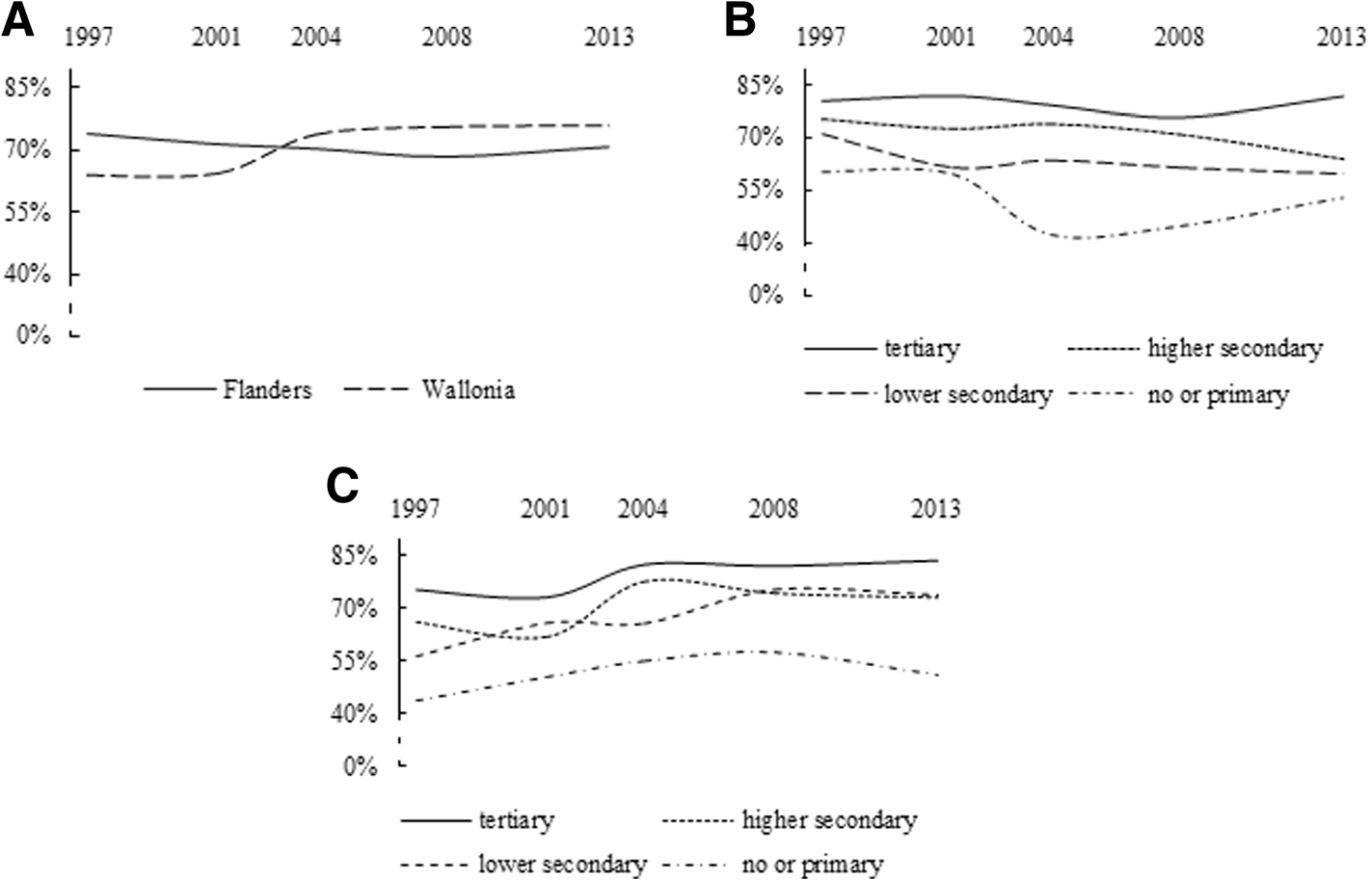
**a** Diffusion of Pap smear use in women 25–64 years old between 1997 and 2013, by region. **b** Diffusion of Pap smear use in women 25–64 years old between 1997 and 2013 in Flanders, by educational level. **c** Diffusion of Pap smear use in women 25–64 years old between 1997 and 2013 in Wallonia, by educational level

As shown in Table 1 and Fig. 1a and Fig. 2a, the diffusion patterns of mammogram and Pap smear use exhibited strong differences. The diffusion pattern of mammogram use was more in line with the diffusion pattern predicted by DOI theory (Fig. 1a). More specifically, between 1997 and 2013, mammogram use clearly became increasingly widespread within the population of women between the ages of 50 and 69 years. This pattern was particularly visible in Flanders, where mammogram coverage increased from 45.6 to 78.1% of the women reporting having had a mammogram in the past two years, although it was also observed in Wallonia, where there was an increase from 51.2 to 69.7%. In Wallonia, however, following a continuous rise since 1997, coverage declined from 76 to 69.7% between 2008 and 2013. In contrast, the diffusion pattern of Pap smear use reflected hardly any change in use over time in either region (Fig. 2a). In Flanders, there was a slight decrease in the number of women between the ages of 25 and 64 years who reported having had a Pap smear in the past three years (from 74 to 70.8%), while Wallonia exhibited a slight increase (from 64.1 to 76%).

**Table 1:** Self-reported mammogram use in women 50–69 years old within the past two years and self-reported Pap smear use in women 25–64 years old within the past three years in Flanders and Wallonia, overall and by educational level, across the five waves of the BHIS (1997-2013)

	1997		2001		2004		2008		2013	
	N	%	N	%	N	%	N	%	N	%
Mammogram use										
Flanders										
Overall	164	45.6	228	53.5	313	69.2	314	71.6	298	78.1
By educational level										
None or primary	46	38.2	61	46	64	61.2	56	62.3	36	66.1
Lower secondary	45	40.2	78	60.7	81	66.3	84	77.4	61	77.5
Higher secondary	42	47.1	56	53.1	102	75.5	90	68.2	119	77.3
Tertiary	31	71	33	56.3	66	73.7	84	78.1	82	85.8
Wallonia										
Overall	189	51.2	287	64	372	73	280	76	285	69.7
By educational level										
None or primary	61	42.5	79	54.3	79	56.2	50	67.4	36	50.6
Lower secondary	50	49.5	92	67.3	92	68.8	72	77.6	37	79.5
Higher secondary	42	62.1	57	64.3	92	81.4	84	76.8	87	75.6
Tertiary	36	57.2	59	75.9	109	84.9	74	79.3	95	68.5
Pap smear use										
Flanders										
Overall	660	74	709	71.5	693	70.4	584	68.5	553	70.8
By educational level										
None or primary	85	60.4	80	60	44	42.4	44	44.9	22	53
Lower secondary	140	71.1	131	61.4	118	63.4	85	61.3	63	59.5
Higher secondary	223	75.5	243	72.7	257	74	201	71	190	63.9
Tertiary	212	80.5	255	81.9	274	79.4	254	75.9	278	81.9
Wallonia										
Overall	546	64.1	669	64.5	790	73.9	608	75.7	589	76
By educational level										
None or primary	60	44	76	50.6	77	55.1	48	57.6	31	51.2
Lower secondary	126	56.6	168	66.1	152	65.9	106	76.6	77	74
Higher secondary	175	66.1	191	61.8	250	77.6	211	74.3	196	73
Tertiary	188	75.1	234	73	311	82.3	243	81.9	285	83.4

Within each cancer screening test, diffusion patterns varied according to educational level, as shown in Fig. 1b, c and Fig. 2b, c. In general, in both regions, women with higher levels of education showed a higher rate of mammogram and Pap smear use than did their counterparts with less education. The results revealed differences with regard to developments in educational disparity in mammogram and Pap smear use. With regard to mammogram use, educational disparities transformed over time, seemingly under the influence of regional screening policies. In Flanders, before the introduction of the national screening programme, considerably more women with high levels of education reported having had a mammogram in the past two years, as compared to those with low levels of education (71% vs. 38.2%), although this disparity decreased after 2001 (Fig. 1b). Surprisingly, this narrowed gap was initially due to reduced mammogram use amongst women who had completed tertiary education, combined with increased use by women with less education. In Wallonia, where the programme was implemented in 2002, different diffusion patterns by educational level were observed (Fig. 1c). In contrast to the situation in Flanders, the introduction of the programme was initially accompanied by a widened education gradient in mammography use. After 2004, however, the gap gradually narrowed, due to decreases in use amongst women with both the highest (from 84.9% in 2004 to 68.5% in 2013) and lowest (from 67.4% in 2008 to 50.6% in 2013) levels of education. Unlike the developments observed in the diffusion patterns of mammogram use, except for a few slight fluctuations, Pap smear use remained quite stable over time, regardless of women’s educational level or region (Fig. 2b and c). This nevertheless implies that, in both regions, the education gradient in Pap smear use has also changed very little over time.

### Development of absolute and relative educational inequalities in mammogram and Pap smear use

**Fig. 3:**
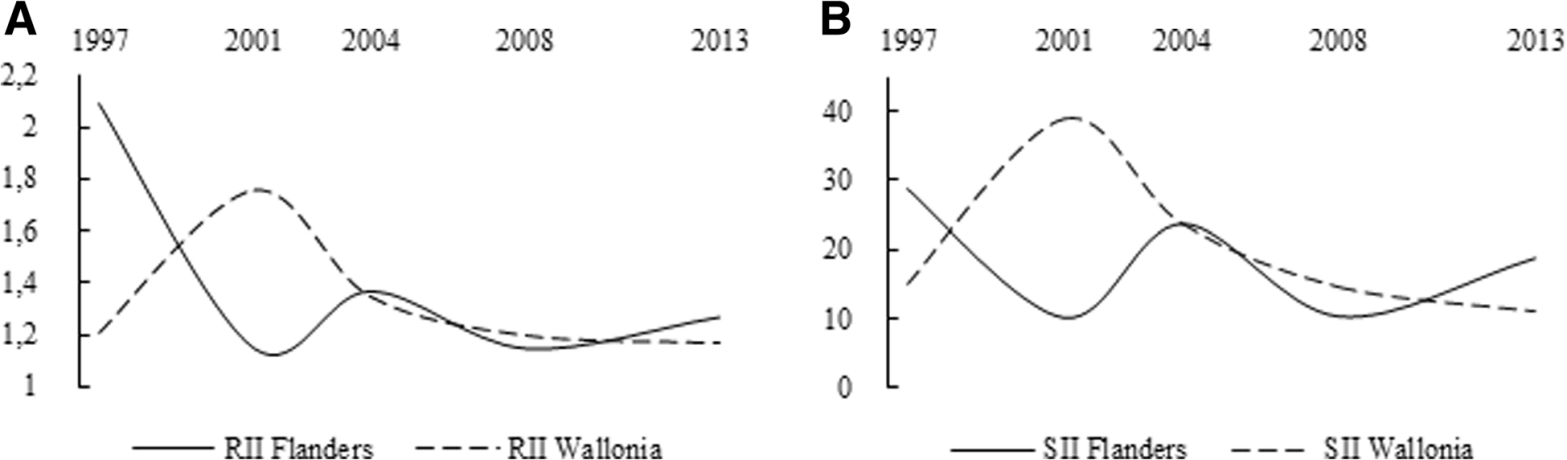
**a** Relative index of inequality (RII) for mammogram use, by region and survey year. **b** Slope index of inequality (SII) for mammogram use, by region and survey year

In a subsequent step, we investigated the statistical significance of changes in absolute and relative inequalities in mammogram and Pap smear use, both successive and overall. Despite the national screening programme for breast cancer, changes in educational inequalities in mammogram use varied considerably by region. In Flanders, relative educational inequalities in mammogram use decreased significantly by 45% (*p* = 0.04) between 1997 (RII = 2.09, 95% CI 1.32–3.31) and 2001 (RII = 1.15, 95% CI 0.84–1.37), when the national screening programme was launched (Table 2). This decline is also clearly visible in Fig. 3a. The overall decrease of relative educational inequalities by 39.2% between 1997 and 2013 was also significant (*p* = 0.05). Despite this significant decline, in 2013, mammogram use in Flemish women with high levels of education was still 1.27 (95% CI 1.03–1.56) times higher than the rate observed amongst their counterparts with less education. In Wallonia (Fig. 3a and b), the education gradient in mammogram use increased between 1997 and 2001 (in contrast to the decrease observed in Flanders during this period), although the disparities also started to decline in Wallonia. It is important to note that, in 1997, absolute and relative inequalities were much lower in Wallonia than they were in Flanders. As demonstrated by the significance tests, however, neither the observed successive changes nor the overall change in educational inequalities were significant in Wallonia (Table 2). These results suggest that, in Flanders, where women are more loyal to the national breast cancer screening programme, the education gradient decreased significantly after the implementation of the programme, while it did not change significantly in Wallonia, where women are more likely to be screened outside the programme in an opportunistic manner.

**Table 2:** Development in the relative index of inequality (RII) and the slope index of inequality (SII) ^a^ for mammogram and Pap smear use in women 50–69 and 25–64 years old, respectively, in Flanders and Wallonia, across the five waves of the BHIS (1997–2013)

	RII	(95% CI)		SII	(95% CI)		RII	(95% CI)		SII	(95% CI)	
	Mammogram use in Flanders (N = 2075)						Mammogram use in Wallonia (N = 2119)					
1997	2.09	(1.32–3.31)	**	28.90	(10.94–48.65)	**	1.21	(0.84–1.75)		15.02	(−6.67–36.71)	
2001	1.15	(0.84–1.37)		10.19	(−10.06–30.44)		1.76	(1.33–2.34)	***	38.82	(20.69–56.95)	***
2004	1.37	(1.07–1.75)	*	23.84	(6.48–41.19)	**	1.35	(1.11–1.66)	**	23.65	(8.33–38.98)	**
2008	1.15	(0.92–1.43)		10.39	(−6.51–27.3)		1.2	(0.96–1.5)		14.55	(−2.26–31.37)	
2013	1.27	(1.03–1.56)	*	18.86	(1.98–35.74)	*	1.17	(0.89–1.55)		11.03	(−11.83–32.24)	
Successive changes	Change	p-value ^b^		Change	p-value ^b^		Change	p-value ^b^		Change	p-value ^b^	
2001 vs. 1997	−45.0%	0.04	*	− 64.7%	0.18		45.5%	0.1		158.5%	0.1	
2004 vs. 2001	19.1%	0.42		133.9%	0.32		−23.3%	0.11		−39.1%	0.21	
2008 vs. 2004	−16.1%	0.28		−56.4%	0.28		−11.1%	0.44		−38.5%	0.44	
2013 vs. 2008	10.4%	0.51		81.5%	0.49		−2.5%	0.89		−24.2%	0.8	
Overall change	Change	p-value ^b^		Change	*p*-value ^a^		Change	p-value ^b^		Change	p-value ^b^	
2013 vs. 1997	−39.2%	0.05	*	−34.7%	0.44		−3.3%	0.88		−26.6%	0.8	
	Pap smear use in Flanders (N = 4405)						Pap smear use in Wallonia (N = 4583)					
1997	1.19	(1–1.41	*	12.42	(−0.36–25.2)		1.46	(1.19–1.8)	***	23.95	(10.26–37.65)	***
2001	1.25	(1.06–1.48)	**	15.28	(2.57–28)	*	1.64	(1.37–1.98)	***	33.62	(21.14–46.1)	***
2004	1.39	(1.16–1.66)	***	25.03	(11.74–38.32)	***	1.38	(1.19–1.6)	***	23.71	(12.55–34.87)	***
2008	1.25	(1.03–1.53)	*	18.22	(3.55–32.88)	*	1.18	(1.01–1.39)	*	12.77	(0.39–25.16)	*
2013	1.58	(1.3–1.92)	***	32.28	(19.78–44.7)	***	1.2	(1.01–1.42)	*	13.34	(0.01–26.67)	*
Successive changes	Change	p-value ^b^		Change	p-value ^b^		Change	p-value ^b^		Change	p-value ^b^	
2001 vs. 1997	5.0%	0.68		23.0%	0.76		12.3%	0.41		40.4%	0.31	
2004 vs. 2001	11.2%	0.4		63.7%	0.3		−15.9%	0.15		−29.5%	0.25	
2008 vs. 2004	−10.1%	0.45		−27.2%	0.5		−14.5%	0.17		−46.1%	0.2	
2013 vs. 2008	26.4%	0.05	*	77.2%	0.15		1.7%	0.92		4.4%	0.95	
Overall change	Change	p-value ^b^		Change	p-value ^b^		Change	p-value ^b^		Change	p-value ^b^	
2013 vs. 1997	32.8%	0.03	**	159.9%	0.03	*	−17.8%	0.14		−44.3%	0.28	

^a^RII and SII values are adjusted for age, country of birth, urbanisation, employment status, necessity of postponing medical consumption and difficulty contributing to healthcare

^b^p-value of the t-test of Altman and Bland on a difference between measures of two different survey years

* *p* < .050; ** *p* < .010; *** *p* < .001

**Fig. 4:**
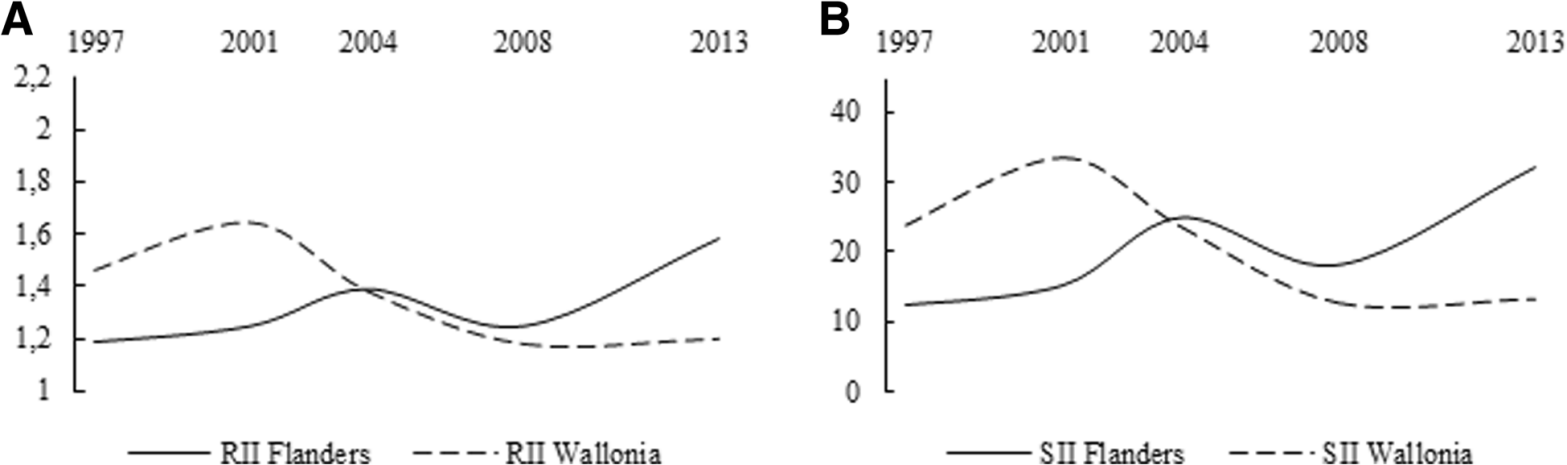
**a** Relative index of inequality (RII) for Pap smear use, by region and survey year. **b** Slope index of inequality (SII) for Pap smear use, by region and survey year

As shown in Table 2, educational inequalities in Pap smear use were large and persistent in both regions between 1997 and 2013. In Flanders, RII increased significantly by 26.4% (*p* = 0.05) between 2008 and 2013. Moreover, there was an overall significant increase of 159.9% (*p* = 0.03) in absolute inequalities between 1997 and 2013, with relative inequalities increasing by 32.8% (p = 0.03). In Wallonia, the observed fluctuations (Fig. 4a and b) in RII and SII between 1997 and 2013 were not significant.

## Discussion

Using repeated cross-sectional data from five successive waves (1997–2001–2004-2008-2013) of the BHIS, this study examines the diffusion patterns of mammogram and Pap smear use in Belgium between 1997 and 2013, in addition to exploring the possible impact of regional screening policies in Flanders and Wallonia on the development of educational inequalities in mammogram and Pap smear use over time.

One finding with respect to the diffusion of Pap smear use was that the proportion of women who indicated that they had been screened in the past three years hardly changed at all over time. On average, coverage was relatively high (70%), although it was still below the generally accepted European target of 75%. This target was reached only in Wallonia, with a coverage of 76% in 2013. Second, in both Flanders and Wallonia, where Pap smear tests were offered predominantly in an opportunistic manner, we found large and persistent educational inequalities in Pap smear use between 1997 and 2013. This finding is in line with previous studies that have highlighted the disadvantages of opportunistic screening in terms of efficiency, quality and equal use [6, 7, 9, 20, 32, 33]. On the one hand, the relatively high rate of Pap smear coverage in Flanders and Wallonia indicates a reasonable degree of spread throughout the population. On the other hand, between 1997 and 2013, consistently more women with high than with low levels of education reported having had a Pap smear in the past three years. This finding is in contradiction to the diffusion pattern predicted by DOI theory. Based on this theory, we expected that Pap smears would initially be accessed by women with higher levels of education, causing an increasing gap in use, which would eventually narrow as the screening test became increasingly widespread within the population and people with lower levels of education caught up with their more highly educated counterparts [48].

The deviation from the diffusion pattern predicted by DOI theory might have been due to a common phenomenon within the context of opportunistic screening that also occurs in Belgium: the combination of over-screening and unequal screening, due to under-screening amongst a portion of the eligible women [30, 31]. In every survey year between 1997 and 2013, women with lower levels of education were less likely to report having had a Pap smear in the past three years, as compared to women with higher levels of education. This pattern was observed in both Flanders and Wallonia. Moreover, the efforts of both regional governments to address the problem of over-consumption by gradually reducing the reimbursement of Pap smears to once every three years did not seem to have paid off in Flanders. On the contrary, according to our results, both absolute and relative educational inequalities in Pap smear use in Flanders increased significantly between 1997 and 2013, with the greatest increase in relative inequality occurring between 2008 and 2013. In Flanders, therefore, the reduced reimbursement of Pap smears primarily affected less-educated women, amongst whom Pap smear use had clearly decreased in the three preceding years.

With regard to the diffusion of mammogram use, our findings were in line with DOI theory, with this screening test clearly becoming increasingly widespread within the eligible population over time (between 1997 and 2013) in both Flanders and Wallonia. In Wallonia, however, mammogram use increased only until 2008, after which it exhibited a remarkable decline. As revealed by an outline of the diffusion patterns by educational level for Wallonia, this decline could be attributed to a decrease in mammogram use in the preceding two years by women with tertiary education, as well as by those who had completed no more than primary education. The growing controversy over mammography screening may have played a role in the declines revealed in our results. Despite European guidelines, mammography screening continues to be a widely debated prevention strategy [49]. More specifically, the debate reflects a consideration between the harmful effects at the individual level (e.g. pain when the mammogram is taken [50], fear and psychological distress caused by false positives [51] and over-diagnosis and over-treatment of breast cancers that would otherwise not be diagnosed during a woman’s life [49, 52]) and the benefits at the population level (e.g. a reduction of 20–30% breast cancer mortality amongst women between the ages of 50 and 69 years [53, 54]). It is possible that the mammography controversy is more pronounced in the south of Belgium, where the greater prevalence of opportunistic screening might increase the likelihood that information on this issue is disseminated by gynaecologists and general practitioners. In Wallonia, gynaecologists play the most important role in encouraging women to have mammograms, while general practitioners are crucial for women with low levels of education [55]. Moreover, perceptions of ambiguity regarding cancer screening have been associated with diminished uptake [56–59]. In particular, people with lower levels of education have been found to exhibit higher levels of ambiguity aversion (i.e. when ambiguity is high, they pessimistically appraise the risks and benefits of action and avoid decision-making) [57, 60, 56]. As suggested by Han and colleagues [60], higher educational attainment might affect perceived ambiguity by enhancing the capacity of individuals to make sense of conflicting health information. In the same vein, Mirowsky and Ross [61] report that people with higher levels of education tend to have a greater capacity to make sense of conflicting health information. Results from a study conducted in the Netherlands amongst women who were invited for breast cancer screening for the first time do indeed suggest higher levels of sufficient knowledge and informed choice in women with higher levels of education [62]. It is therefore plausible that, in Wallonia, the reduction in mammogram use amongst more highly educated women resulted from informed and conscious choices, with ambiguity aversion playing a greater role in the diminished use amongst women with less education.

At the outset of this study, we suggested that government policies (and, more specifically, the introduction of the national breast cancer screening programme in 2001 in Flanders and in 2002 in Wallonia) could have had an impact on the diffusion of mammogram use and the associated educational inequalities. In light of previous research noting that most Walloon women are screened in an opportunistic manner outside the screening programme during consultations with their general practitioners or gynaecologists, we also expected that the different screening climates of Flanders and Wallonia would be reflected in different patterns of diffusion. According to our results, in 1997, educational inequalities were lower in Wallonia than they were in Flanders. By charting developments over time, however, it became clear that, in Wallonia, despite the implementation of the national screening programme in 2002, educational inequalities in mammogram use did not change significantly between 1997 and 2013. In contrast, in Flanders, educational inequalities decreased significantly between 1997 and 2001, when the screening programme was launched, with an overall decline being observed between 1997 and 2013. Consistent with our assumptions, the national breast cancer screening programme in Flanders advanced the diffusion of mammogram use within the population and reduced, although it did not eliminate the educational inequalities in mammogram use, while this was not the case in Wallonia.

### Limitations

This study provides valuable new insight into the impact of regional screening policies on the development of educational inequalities in Pap smear and mammogram use in Belgium. It is nevertheless subject to several limitations. First, the health-interview data on which the study is based have several weak points. Mammogram and Pap smear use are based on self-reported information, which might be subject to recall bias, and particular to an overestimation of use, as people tend to underestimate the length of time since they last had a mammogram or Pap smear. Comparison with data from the National Institute for Social Security has indeed demonstrated that the BHIS data overestimate the overall coverage of mammogram and Pap smear use [55]. Nevertheless, the BHIS remains an important source of information, as it offers the unique opportunity to examine developments in cancer screening coverage over time, as well as the relationship of such developments with several socio-economic parameters, which are not available or suitable in the data from the National Institute for Social Security. Moreover, linking of the 2008 BHIS data to data from the insurance institutions has demonstrated that the validity of the BHIS data (in terms of sensitivity and specificity) does not vary significantly by level of education. Another weak point of the BHIS is its lack of detailed information regarding non-participation. This prevented us from assessing bias related to socioeconomic status. Previous research on health-interview data has indicated that lower socioeconomic groups tend to have a higher non-response rates on items relating to cancer screening and subjective health [63]. The data used in this study might therefore underestimate inequalities in mammography and Pap smear use with regard to poorer uptake amongst lower socioeconomic groups.

Second, although the repeated cross-sectional research design made it possible to draw conclusions on how the use of screening tests amongst women with high and low levels of education have changed over time, it hampered the causal interpretation of the results. Nonetheless, previous studies have provided compelling evidence of a causal relationship running from more schooling to better health [64–66]. Additionally, in the current study, all outcomes occurred after the respondents had completed their education.

Third, we advanced educational attainment as the main mechanism of stratification, as the knowledge and skills acquired during education could make people more receptive to health information, in addition to making them better equipped to obtain these messages and access appropriate health services [61]. Moreover, in contrast to other stratification indicators (e.g. type of employment and income), educational level is more stable over time, and information on this characteristic is largely available for almost all of the women in the study. Our statistical models nevertheless accounted for indicators relating to employment and income by including work status, as well as the necessity of postponing medical consumption and difficulties in contributing to healthcare. In addition, and similar to the study by Renard and colleagues [5], a sensitivity analysis using income level (available only at the household level) as an indicator of stratification did not demonstrate the same effects, nor did it reveal any clear time-pattern of the inequalities. In addition to the higher level of missing answers (*N* = 507; 13.7%), it is plausible that household income level captures aspects of women’s social position other than their individual educational level, and that those indicators are not interchangeable. This should be investigated more in detail, but doing so would have exceeded the scope of this study.

Fourth, given that this study focuses only on Belgium, the generalisability of the results could be questioned. This study thus paves the way for future studies investigating whether our findings also apply in other national contexts.

## Conclusions

We can conclude that, with regard to cancer screening in Belgium, diffusion patterns vary by cancer screening strategy and regional screening policy. In the case of opportunistic screening, which was the strategy for offering Pap smears in Flanders and Wallonia between 1997 and 2013, diffusion patterns did not reflect those predicted by DOI theory. An outline of diffusion patterns according to educational level reveals that the relatively large extent of Pap smear use in Flanders and Wallonia between 1997 and 2013 was accompanied by consistently high educational inequalities in use over time. The results of this study thus emphasise the potential drawbacks of opportunistic screening in terms of over-screening (i.e. well diffused use within the eligible population, albeit only in the highly educated segment, due to their excessive screening behaviour).

By highlighting the impact of regional screening policies, this study demonstrates that the diffusion of cancer screening can contribute to equality in participation over time, under the influence of an organised screening programme, but only if the programme occupies a prominent place amongst the available screening options. As revealed by our examination of the Belgian case, a screening programme cannot be efficient and reduce or eliminate educational inequalities in screening unless it is promoted by and conducted in cooperation with general practitioners and gynaecologists. Despite its national character, Flanders and Wallonia approached their organised breast cancer screening programmes differently, and these differences were reflected in differences in the development of educational inequalities in mammogram use. This calls the effectiveness of the Belgian programme into question. More specifically, although the screening programme in Flanders certainly has the potential to reduce educational inequalities in use, additional effort is needed in order to eliminate all disparities between women with low and high levels of education. The findings reveal a different situation in Wallonia, where widespread opportunistic screening competed with the organised screening programme. This apparently impeded the effective functioning of the organised programme, as well as its positive impact on the education gradient in mammogram use.

In summary, by applying DOI theory to inequalities in cancer screening in a regionally variating context, this study highlights two aspects that should be addressed in future research: [1] the possibility that, in the case of an opportunistic screening strategy, widespread use can imply an underlying problem of over-screening and associated disparities in use, and [2] the possibility that regional variations in support of a national screening programme can result in regional variation in patterns of diffusion for cancer screenings, as well as in the development of inequalities in use.

## Abbreviations

BHIS: Belgian Health Interview Survey
CI: Confidence interval
DOI: Diffusion of innovation theory
ISCED: International Standard Classification of Education
RII: Relative index of inequality
SII: Slope index of inequality
